# The Type of Preoperative Oral Antithrombotics as a Risk Factor for Venous Thromboembolism After Hip Surgery: A Retrospective Study

**DOI:** 10.3390/medicina61040729

**Published:** 2025-04-15

**Authors:** Yea-Ji Lee, Jaemoon Lee, Seung-Wan Hong, Seong-Hyop Kim

**Affiliations:** 1 Department of Anaesthesiology and Pain Medicine, Konkuk University Medical Centre, Seoul 05030, Republic of Korea; ladydaisy82@naver.com (Y.-J.L.); resist85@gmail.com (J.L.); 20180077@kuh.ac.kr (S.-W.H.); 2 Department of Infection and Immunology, School of Medicine, Konkuk University, Seoul 05030, Republic of Korea; 3 Research Institute of Medical Science, School of Medicine, Konkuk University, Seoul 05030, Republic of Korea

**Keywords:** antithrombotics, hip surgery, postoperative complications, venous thromboembolism

## Abstract

*Background and Objectives*: Hip surgery is increasingly performed among elderly patients. Oral antithrombotics, which are taken for patients’ underlying diseases, are a main concern regarding perioperative bleeding. Postoperative venous thromboembolism (VTE) is a leading cause of mortality after hip surgery. Therefore, administration of preoperative oral antithrombotics is a double-edged sword in hip surgery. In this study, we examined the correlation between the occurrence of postoperative VTE and the type of oral antithrombotics administered preoperatively. *Materials and Methods*: We analyzed the medical records of 601 patients aged 19 and over who underwent hip surgery from January 2021 to June 2023. The patients were assigned to two groups as follows: Groups VTE+ (patients who developed postoperative VTE) and VTE- (patients who did not develop postoperative VTE), respectively. *Results*: Of the 139 patients who had been taking oral antithrombotics for 6 months or more, 24 were allocated to group VTE+ and 115 to group VTE-, respectively. The number of patients who took clopidogrel and cilostazol was significantly higher in groups VTE- and VTE+, respectively (12.5 vs. 33.9%, *p* = 0.038, odds ratio (OR) = 0.278, 95% confidence interval (CI) = 0.078–0.991; 20.8 vs. 5.2%, *p* = 0.010, 95% CI = 1.325–17.245; group VTE+ vs. group VTE-). Preoperative albumin levels were significantly lower in group VTE+ (3.4 ± 0.6 g/dL vs. 3.7 ± 0.4 g/dL, *p* = 0.004, OR = 0.285, 95% CI = 0.115–0.702). In multivariate regression analysis, the results were statistically significant for clopidogrel, cilostazol, and preoperative albumin levels (*p* = 0.035, OR = 0.237, 95% CI = 0.062–0.901; *p* = 0.011, OR = 6.479, 95% CI = 1.542–27.226; *p* = 0.002, OR = 0.211, 95% CI = 0.080–0.558). *Conclusions*: Among the patients who had been taking oral antithrombotics for ≥6 months, clopidogrel had a prophylactic effect, but cilostazol showed an aggravating effect on postoperative VTE in hip surgery. Preoperative hypoalbuminemia increases the risk of postoperative VTE in hip surgery.

## 1. Introduction

Hip surgery is performed to treat conditions such as traumatic fractures, rheumatoid arthritis, avascular necrosis, and osteoarthritis (OA). In modern societies, femoral neck or intertrochanteric fractures and degenerative OA are prevalent among older adults [1,2]. In 2019, approximately 14.2 million cases of hip fractures were reported [3]. Moreover, approximately 10% of individuals aged > 45 years have radiographic signs of hip OA, and 93% of procedures are performed for severe OA, which is characterized by intractable pain and functional limitations, leading to an increased number of patients undergoing hip surgery [4,5]. This increasing trend is accompanied by an elevation in associated complications, including venous thromboembolism (VTE).

VTE is a potentially life-threatening complication. The risk of postoperative VTE, including deep vein thrombosis (DVT) and pulmonary thromboembolism (PTE), is substantial [6,7]. In addition, PTE is the primary cause of mortality among postoperative complications [8]. This can lead to an increased length of hospital stay in the short term and an increased duration of intensive care unit admission, ultimately contributing to higher long-term mortality rates [7,9,10].

Prescription of direct oral anticoagulants (DOACs) has rapidly increased for the prevention of embolic stroke with atrial fibrillation and for the prevention and treatment of VTE [11,12]. However, the interruption of perioperative antithrombotics is required due to increased bleeding risk in some high-risk patients. There are several guidelines for the perioperative management of antithrombotics, including aspirin (which irreversibly inhibits cyclooxygenase-1, required to make thromboxane precursors in platelets), warfarin (which blocks the enzyme that activates vitamin K, reducing the production of clotting factors), low-molecular weight heparin (which binds to antithrombin and increases antithrombin-mediated inhibition of the synthesis and activity of coagulation factor Xa) and DOACs (which inhibit factor Xa or thrombin) [13]. These practical guidelines suggest when to stop and resume each categorical agent [14]. However, there are not enough studies conducted to evaluate how each agent and the duration of interruption affect postoperative VTE.

In this study, we aimed to demonstrate the correlation between the type of preoperative oral antithrombotics and postoperative VTE. We also assessed whether the duration of antithrombotics interruption affected the incidence of postoperative VTE.

## 2. Materials and Methods

This study was approved by the Institutional Review Board of the Konkuk University Medical Center (approval number: KUH2024-09-043). The review of electronic medical records and data extraction from our center’s clinical data warehouse were conducted by one of our co-investigators. All data were saved and collected anonymously. The Strengthening the Reporting of Observational Studies in Epidemiology (STROBE) checklist guided the reporting of this study (see the Supplementary Material S1).

### 2.1. Patient Inclusion and Exclusion Criteria

Patients aged > 19 years who underwent hip surgery at the Konkuk university medical center (tertiary referral hospital) from January 2021 to June 2023 were selected. All hip surgeries, including internal and external fixation, partial hip joint replacement (hemiarthroplasty), and total hip joint arthroplasty on the femur, hip joint, and pelvic bone, were performed by the same orthopedic surgeon. The exclusion criteria were as follows: (1) administration of preoperative oral antithrombotics: not at all or for less than 6 months; (2) inability to proceed with surgery; (3) reoperation during hospitalization; (4) absence of preoperative or postoperative computed tomography (CT) images of the chest or lower extremities; (5) preoperative administration of heparin; and (6) diagnosis of VTE before surgery. Following the selection, the patients were allocated to two groups: Groups VTE+ (developed postoperative VTE) and VTE- (did not develop postoperative VTE), respectively.

### 2.2. Anesthetic Procedures

Patients underwent hip surgery under either general or spinal anesthesia. The type of anesthesia was selected at the discretion of the anesthesiologist. When spinal anesthesia was converted to general anesthesia, it was classified as general anesthesia. The anesthetic techniques were performed according to the standardized protocol of our center.

In all cases of anesthesia, routine monitoring of pulse oximetry, non-invasive arterial pressure, and electrocardiogram was started before induction of anesthesia.

General anesthesia was conducted as follows:

Preoxygenation with 100% oxygen was applied through a mask. For the induction of anesthesia, 2 mg/kg of propofol was administered intravenously. After the loss of consciousness, mask ventilation was performed with oxygen and an inhalational agent (sevoflurane). As mask ventilation was confirmed, intravenous 0.6 mg/kg of rocuronium (for neuromuscular blockade) and target-controlled infusion (TCI) of remifentanil were administered. When hemodynamic stability was achieved, tracheal intubation was performed and mechanical ventilation was applied. During the surgery, anesthesia was maintained with inhaled sevoflurane and TCI of remifentanil. At the end of surgery, the administration of sevoflurane and remifentanil was stopped, and residual neuromuscular blockade was reversed with intravenous 2–4 mg of sugammadex. After tracheal extubation, the patient was transferred to the post-anesthetic care unit.

Spinal anesthesia was conducted as follows:

A facial mask was applied to all patients for oxygenation. The patient assumed the lateral decubitus position with the operated hip facing downward. The skin of the patient’s back was prepared using aseptic packets of chlorhexidine. After identifying the lumbar spinous processes of L3, L4, and L5 by direct palpation, a 25-gauge spinal needle was inserted into the interspace between two spinous processes at the chosen spinal level. If cerebrospinal fluid flowed well from the needle hub, heavy bupivacaine was administered intrathecally. Once adequate blockade height was achieved, the patient was positioned for surgery. The patient was sedated with an intravenous infusion of dexmedetomidine or remimazolam during the surgery. At the end of surgery, the sedative was discontinued, and after the patient’s respiration was secured, the patient was transferred to the post-anesthetic care unit.

### 2.3. Diagnosis of VTE

According to our center’s protocol, chest and lower extremity CT scans for PTE and DVT were performed before and after the hip surgery. Prior to the CT scanning, the surgeon provided the patient with information, including about the surgical process, perioperative risks, postoperative complications, and the purpose of CT scanning. Informed consent was obtained from the patient.

The diagnosis of PTE or VTE was determined by the same vascular and interventional radiologist at our center as follows: (1) a filling defect found on chest CT scan (PTE) or (2) a filling defect found on lower extremity CT scan (DVT).

### 2.4. Use of Antithrombotics

The antithrombotics used included aspirin, clopidogrel, cilostazol, DOACs, and warfarin. Heparin was not included in the antithrombotics because it was used for the treatment of DVT or PTE; however, it was not used as a preoperative medication.

### 2.5. Interruption of Antithrombotics Before Surgery

The interrupted duration followed the generally recommended protocol [14,15]: aspirin (7 days), clopidogrel (5 days), cilostazol (2–3 days), DOACs (1–4 days), and warfarin (2 days). The duration is categorized into three groups: ‘under’ (shorter duration than that of recommended protocol), ‘normal’ (duration within recommended protocol), and ‘over’ (longer duration than that of recommended protocol).

### 2.6. Preoperative Labratoy Examinations

According to our center’s protocol, laboratory examinations including complete blood count, blood chemistry, coagulation panel, and D-dimer were performed within 1 month of surgery.

### 2.7. Statistical Analysis

The primary outcome was the incidence of postoperative VTE for each antithrombotic agent. The secondary outcome was the incidence of postoperative VTE for each categorized interruption duration of antithrombotics.

Statistical analysis was conducted by our co-investigator using Statistical Package for the Social Sciences software (ver. 29.0; IBM Corp., Armonk, NY, USA). Categorical variables were analyzed using the chi-squared or Fisher’s exact test, whereas continuous variables were analyzed using the independent t-test. Pearson’s correlation coefficient was used to assess the correlation between the demographic data of continuous variables and postoperative VTE. Categorical data was analyzed using logistic regression analysis to identify potential risk factors for postoperative VTE. Factors with *p* < 0.05 in the regression analysis were incorporated in a multivariate conditional logistic regression model utilizing backward stepwise regression procedures. Data are presented as the number of patients (%), mean ± standard deviation (SD), or median (range). Statistical significance was set at *p* < 0.05.

## 3. Results

**Figure 1:**
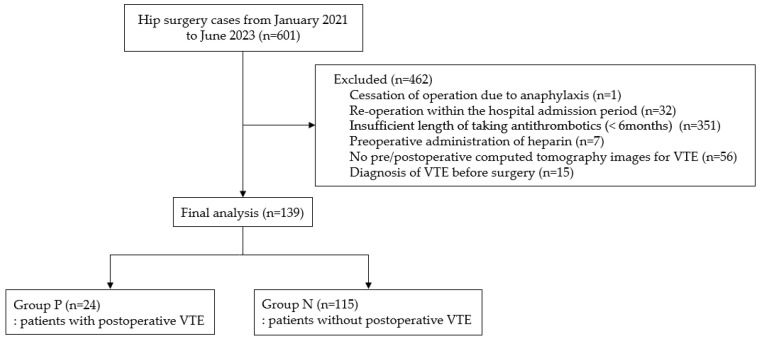
Details of the patient selection process.

In this study, we reviewed the EMR of 601 patients. However, we excluded one patient due to cessation of the operation (anaphylaxis due to anesthetic agents), 32 on account of reoperation during hospitalization, 351 due to insufficient length of taking antithrombotics (having not taken or having taken them for less than 6 months), 7 due to preoperative administration of heparin, 56 due to lack of pre- or postoperative CT images for PTE or VTE, and 15 due to a diagnosis of PTE or VTE before hip surgery. Of 139 patients, 24 and 115 were allocated to Groups VTE+ and VTE-, respectively (Figure 1).

### 3.1. Characteristics of the Patients

Demographic data are shown in Table 1. The patient heights were significantly shorter in group VTE+ (154.0 [8.0] vs. 160.0 [12.0], *p* = 0.036).

### 3.2. Use of Antithrombotics and Preoperative Laboratory Results (Table 2)

The number of patients who took clopidogrel was significantly higher in group VTE-; however, the number of patients who took cilostazol was significantly higher in group VTE+ (12.5 vs. 33.9%, *p* = 0.038, 95% CI = 0.078–0.991; 20.8 vs. 5.2%, *p* = 0.010, 95% CI = 1.325–17.245). Preoperative albumin level showed a significant difference between the two groups (34.0 ± 0.6 g/dL vs. 3.7 ± 0.4 g/dL, *p* = 0.004). The interrupted duration of antithrombotics did not significantly increase the incidence of VTE.

**Table 2:** Status of antithrombotics use and laboratory findings.

	Group VTE+ (*n* = 24)	Group VTE- (*n* = 115)	*p* Value	95% CI
Antithrombotics, *n* (%)				
Aspirin	13 (54.2)	61 (53.0)	0.920	0.433–2.529
Clopidogrel	3 (12.5)	39 (33.9)	0.038 *	0.078–0.991
DOAC	7 (29.4)	29 (25.2)	0.688	0.460–3.239
Warfarin	0 (0.0)	1 (0.9)	0.647	0.974–1.008
Cilostazol	5 (20.8)	6 (5.2)	0.010 *	1.325–17.245
Interrupted duration, *n* (%)			0.061	
Under	10 (41.7)	72 (62.6)		
Normal	13 (54.2)	34 (29.6)		
Over	1 (4.2)	9 (7.8)		
Preoperative laboratory results				
Platelet (10^3^/μL)	228.0 (112.8)	204.0 (69.3)	0.715	
PT INR	1.0 (0.1)	1.0 (0.1)	0.989	
aPTT (sec)	37.0 ± 4.8	39.4 ± 6.8	0.115	
D-dimer (μg/mL)	7.5 (1.5)	7.1 (1.5)	0.881	
Albumin (g/dL)	3.4 ± 0.6	3.7 ± 0.4	0.004 *	
HbA1c (%)	5.8 (1.7)	5.9 (0.9)	0.326	

Data are presented as numbers (%), median (range) or value ± standard deviation. 95% CI, 95% confidence interval; DOAC, direct oral anticoagulants; D-dimer, fibrin D-dimer; PT INR, prothrombin time-international normalized ratio; aPTT, activated partial thromboplastin time. Interrupted duration: interrupted duration of antithrombotics; ‘under’ (shorter duration than that of recommended protocol); ‘normal’ (duration within recommended protocol); ‘over’ (longer duration than that of recommended protocol). * indicates the results which show *p* value < 0.05.

### 3.3. Regression Analysis for Predicting Risk Factors of VTE (Table 3)

The adjusted ORs were 0.237 and 6.479, with *p* values of 0.035 and 0.011 for clopidogrel and cilostazol, respectively. The adjusted OR was 0.211 (*p* value = 0.002) for the preoperative albumin level. The duration of antithrombotic discontinuation did not differ significantly between the groups.

**Table 3:** Regression analysis of predictors for postoperative VTE.

Variables	Univariable		Multivariable	
	OR	*p* Value	OR	*p* Value
Age	1.057 (0.998–1.119)	0.060		
Height	0.955 (0.903–1.011)	0.112		
Weight	0.978 (0.935–1.022)	0.324		
BMI	1.010 (0.899–1.135)	0.870		
Sex (female/male)	0.840 (0.496–1.423)	0.517		
Anesthetic type (GA/SA)	1.121 (0.249–5.043)	0.882		
Anesthetic time	1.001 (0.993–1.010)	0.733		
Hypertension	0.607 (0.225–1.637)	0.321		
Diabetes	0.465 (0.179–1.207)	0.116		
Atrial fibrillation	1.178 (0.395–3.513)	0.769		
CVA history	0.837 (0.331–2.120)	0.708		
Preoperative delirium	0.209	0.000		
ESRD	3.394 (0.535–21.513)	0.195		
ACEi	0.000 (0.000)	0.999		
ARB	0.749 (0.310–1.810)	0.521		
BB	1.001 (0.991–1.010)	0.894		
CCB	0.606 (0.250–1.468)	0.267		
Diuretics	1.410 (0.528–3.766)	0.494		
Insulin	1.207 (0.129–11.297)	0.869		
Hypoglycemic agents	0.519 (0.191–1.405)	0.197		
Aspirin	1.046 (0.433–2.529)	0.920		
Clopidogrel	0.278 (0.078–0.991)	0.048 *	0.237 (0.062–0.901)	0.035 *
DOAC	1.221 (0.460–3.239)	0.688		
Warfarin	0.000 (0.000)	1.000		
Cilostazol	4.781 (1.325–17.245)	0.017 *	6.479 (1.542–27.226)	0.011 *
Antithrombotics cut duration	1.509 (0.778–2.927)	0.224		
Platelet	1.001 (0.995–1.007)	0.841		
PT INR	0.678 (0.075–6.170)	0.730		
aPTT	0.942 (0.874–1.015)	0.117		
D-dimer	1.000 (1.000–1.001)	0.669		
Albumin	0.285 (0.115–0.702)	0.006 *	0.211 (0.080–0.558)	0.002 *

Data are presented as OR (95% CI). OR, odds ratio; 95% CI, 95% confidence interval. VTE, venous thromboembolism; BMI, body mass index; GA, general anesthesia; SA, spinal anesthesia; CVA, cerebrovascular accident; ESRD, end-stage renal disease; ACEi, angiotensin-converting-enzyme inhibitor; ARB, angiotensin receptor blocker; BB, beta-blocker; CCB, calcium channel blocker; DOAC, direct oral anticoagulants; PT-INR, prothrombin time international normalized ratio; aPTT, activated partial thromboplastin time; D-dimer, fibrin D-dimer. * indicates the results which show *p* value < 0.05.

## 4. Discussion

The present study showed that clopidogrel had a prophylactic effect, but that cilostazol had a provoking effect on postoperative VTE after hip surgery in patients with preoperative antithrombotics. This study also demonstrated that preoperative low serum albumin level was a risk factor for postoperative VTE.

Clopidogrel is primarily used for the prevention of arterial thrombus [16,17]; however, the results showed that it prevents VTE. Clopidogrel irreversibly inhibits the P2Y12 ADP receptor, preventing platelet activation and aggregation. ADP is key in promoting platelet aggregation [16,18,19,20,21]. Clopidogrel reduces platelet adhesion and thrombus formation by blocking ADP receptors [17,18,22]. This mechanism is crucial for the prevention of arterial thrombus; however, it plays a key role in the prevention of venous thrombus [23,24]. The effectiveness of clopidogrel in preventing venous thrombosis has been confirmed in animal models [21]. In animal studies, clopidogrel suppresses endothelial inflammation [19]. In addition, clopidogrel protects endothelial cells, which are essential for regulating blood coagulation, thereby preventing thrombus formation by maintaining its function [17,20,25]. Clopidogrel helps to prevent this damage, which contributes to the prevention of venous thrombus. Clopidogrel promotes fibrinolysis, which is important in preventing the formation and growth of thrombi [16]. Fibrinolytic reactions require both fibrinolytic agents and immune system responses, including neutrophils [23,24]. Clopidogrel acts on neutrophils, promoting their migration to sites of thrombus formation [19,25]. This mechanism may have positive effects on preventing VTE in patients who had been taking antithrombotics due to their concomitant diseases after hip surgery. Additionally, the duration of antithrombotic discontinuation could not be established as a risk factor for VTE in this study. These results would suggest that the type of antithrombotic agent preoperatively used has a greater effect on preventing VTE than the duration of discontinuation.

Cilostazol is an antithrombotic drug that is commonly used in dual or triple antiplatelet regimens in South Korea, Japan, and China [26]. It is often combined with aspirin or aspirin and clopidogrel. Cilostazol inhibits phosphodiesterase, which increases cyclic adenosine monophosphate levels, ultimately preventing platelet aggregation [26,27]. The cilostazol’s negative effect on preventing postoperative VTE observed in this study can be explained by the condition in which cilostazol is mainly administered. Cilostazol is typically utilized for atherosclerotic diseases, where the condition differs from the postoperative VTE. Patients undergoing hip surgery experience prolonged periods of immobilization, pre- and post-operatively, leading to venous stasis, which causes VTE [28,29]. This process predominantly involves the coagulation pathway; the fibrin complex and platelet aggregation play significant roles [23,24]. In addition, cilostazol is not used as a monotherapy for atherosclerosis but rather as an adjunct [26,27,30]. Moreover, unlike other antithrombotic drugs, cilostazol has a reversible effect; therefore, it is not used alone [22,26,27,30]. The discontinuation of cilostazol for surgery may have caused postoperative VTE due to its reversible effects.

Low serum albumin levels are commonly associated with an increased incidence of VTE [31]. This is well known in patients with chronic conditions such as nephrotic syndrome and cancers and in acutely ill hospitalized patients, where the degree of hypoalbuminemia is a significant risk factor for VTE [32,33,34,35,36]. Several studies have reported that low serum albumin (<3.5 g/dL) is a significant risk factor for VTE [31]. Low serum albumin levels contribute to the development of VTE for three primary reasons. First, it indicates poor general health and hyperinflammation [31,36]. Albumin is a powerful antioxidant and reduces vascular inflammation. Low serum albumin levels can cause increased oxidative stress and inflammatory responses. Inflammation can damage the endothelial cells and promote platelet adhesion and aggregation, which increases the risk of thrombus formation. Second, albumin is known to have anticoagulant properties by inhibiting platelet aggregation and fibrin polymerization. Furthermore, albumin shows heparin-like action by increasing the effect of antithrombin [35]. Moreover, hypercoagulability is linked to low serum albumin levels owing to greater levels of fibrinogen and factor VIII [31]. Third, malnutrition influences thrombus formation. Low albumin levels are often associated with malnutrition. Moreover, it can deteriorate vascular health and immune function, thereby increasing the risk of thrombus formation [36].

DOACs and warfarin did not show a positive effect on the prevention of postoperative VTE in this study; however, recent studies reported that they showed comparable efficacy in the treatment and prophylaxis of VTE in high-risk patients [37,38]. Due to their several advantages, DOACs have become the preferred oral antithrombotics, replacing warfarin. Although warfarin carries a higher risk of major bleeding than DOACs, it still has distinct advantages for patients with mechanical heart valves or triple-positive antiphospholipid antibody syndrome [39,40,41].

The duration of interruption of antithrombotics was found to be not associated with the occurrence of postoperative VTE. The interruption of antithrombotics has shown a relatively low risk of thromboembolic events in some studies [42,43]. In contrast, several orthopedic surgery guidelines and studies demonstrated that continuing prophylaxis of VTE did not increase the risk of major bleeding and prevented stroke events effectively [44]. The decision to maintain or discontinue antithrombotics should be accompanied by precise assessment of patients and the risk of the procedures in question.

The patients in Group N were significantly taller. This might be consistent with the findings of a previous study, which stated that patients’ average limb length was longer in the no-thrombus group [45]. However, height was not included as a predictive factor for VTE occurrence in this study.

### Limitations of the Study

First, this study was conducted in a single center with a limited number of patients. Further studies should be carried out with larger numbers of patients for more reliable results. Second, only one patient took warfarin, and the number of patients was therefore not sufficient for statistical significance. Further evaluation would be needed with more patients taking warfarin for the solid determination of the effect of warfarin on the occurrence of VTE. Third, as we collected data from our center’s clinical data warehouse that were established based on electronic medical records, some data could not be collected sufficiently. Representative risk factors for VTE, such as history of VTE, annual caseload per hospitals or facilities, preoperative nutritional status, and reduced mobility [46,47], should also have been analyzed, but such results could not be provided in this study due to insufficient data. The data obtained from larger institutions such as national healthcare insurance services could provide more confirmative results regarding the risk factors for postoperative VTE.

## 5. Conclusions

In this study, valuable information regarding the different effects of each oral antithrombotics on the prevention of postoperative VTE was provided. Specific guidelines have not yet been established; however, this study emphasizes the importance of careful monitoring and selection of oral antithrombotics before hip surgery. Clopidogrel might be helpful in reducing the occurrence of postoperative VTE in hip surgery. It is particularly important to be cautious about the possibility of postoperative VTE in patients with cilostazol or low serum albumin levels. These considerations can help to improve patient outcomes and reduce the incidence of VTE after hip surgery.

## Figures and Tables

**Table 1:** Demographic data.

	Group VTE+ (*n* = 24)	Group VTE- (*n* = 115)	*p* Value	95% CI
Baseline characteristics				
Age (yr)	85.0 (11.0)	81.5 (8.0)	0.058	
Height (cm)	154.0 (8.0)	160.0 (12.0)	0.036 *	
Weight (kg)	54.4 (18.5)	55.2 (14.0)	0.371	
BMI (kg/m^2^)	22.6 ± 5.3	22.5 ± 3.4	0.903	
Female, *n* (%)	18 (75.0)	70 (60.9)	0.19	
Anesthetic type				
General anesthesia, *n* (%)	24 (100.0)	115 (100.0)		
Anesthetic time (min)	180.0 (80.1)	190.5 (63.0)	0.865	
Past medical history, *n* (%)				
Hypertension	17 (70.8)	92 (80.0)	0.321	0.225–1.637
Diabetes	7 (29.2)	54 (47.0)	0.110	0.179–1.207
Atrial fibrillation	5 (20.8)	21 (18.3)	0.769	0.395–3.513
CVA history	8 (33.3)	43 (37.4)	0.708	0.331–2.120
Preoperative delirium	0	0		
ESRD	2 (8.3)	3 (2.6)	0.171	0.535–21.513
Medications, *n* (%)				
Antihypertensives				
ACEi	0 (0.0)	2 (1.7)	0.515	0.959–1.007
ARB	11 (45.8)	61 (53.0)	0.520	0.310–1.810
BB	7 (29.2)	32 (27.8)	0.894	0.405–2.818
CCB	12 (50.0)	71 (62.3)	0.264	0.250–1.468
Diuretics	7 (29.2)	26 (22.6)	0.492	0.528–3.766
Antidiabetic agents				
Insulin	1 (4.2)	4 (3.5)	0.869	0.129–11.297
Hypoglycemic agent	6 (25.0)	45 (39.1)	0.191	0.194–1.405

Data are presented as numbers (%) or median (range). VTE+, developed postoperative venous thromboembolism; VTE-, did not develop venous thromboembolism; 95% CI, 95% confidence interval; BMI, body mass index; CVA, cerebrovascular accident; ESRD, end-stage renal disease; ACEi, angiotensin-converting enzyme inhibitor; ARB, angiotensin receptor blocker; BB, beta-blocker; CCB, calcium channel blocker. * indicates the results which show *p* value < 0.05.

## Data Availability

The data presented in this study are available upon request from the corresponding author, owing to the restrictions imposed by the Institutional Review Board, which approved the study protocol.

## Supplementary Materials

The following supporting information can be downloaded at: https://www.mdpi.com/article/10.3390/medicina61040729/s1, STROBE Statement—checklist of items that should be included in reports of observational studies.
